# Distribution of intra‐host variations and mutations in the genomes of SARS‐CoV‐2 and their implications on detection and therapeutics

**DOI:** 10.1002/mco2.186

**Published:** 2022-12-02

**Authors:** Dongyan Xiong, Xiaoxu Zhang, Junping Yu, Hongping Wei

**Affiliations:** ^1^ CAS Key Laboratory of Special Pathogens and Biosafety Center for Biosafety Mega‐Science Wuhan Institute of Virology Chinese Academy of Sciences Wuhan China; ^2^ CAS Key Laboratory of Special Pathogens and Biosafety University of Chinese Academy of Sciences Beijing China

**Keywords:** adaptive evolution, antiviral drugs, genetic polymorphism, intra‐host diversity, PCR detection, SARS‐CoV‐2 VOCs, vaccine

## Abstract

The ongoing circulation of SARS‐CoV‐2 variants of concern (VOCs) has caused global concerns, because VOCs could escape current vaccines, antiviral drugs, and diagnosis. Analyzing mutations and intra‐host diversities in different and widespread VOCs can provide important insights to virus adaptive evolution and validity of vaccines, antiviral drugs, and diagnosis. In this study, by analyzing 1744 high‐throughput sequencing data for intra‐host single‐nucleotide variations (iSNVs) and 3,668,205 genome sequences for mutations in different VOCs, it was found that Omicron variant is still evolving at high speed, especially having high iSNVs frequency in its S and N genes. The efficacies of antibodies or detection primers targeting these two genes are at high risks to be invalid. Instead, highly conserved regions such as NSP8 gene could be better therapeutic and detection targets. Furthermore, mutations in later VOCs could be traced to the minor alleles in the previous variant samples such as Alpha and Delta in different countries. Finally, it was found that mutations C14408T in RdRp and A18163G in NSP14 gene might be associated with the higher genetic diversity in Omicron. Our findings not only contribute to understanding the adaptive evolution of SARS‐CoV‐2 VOCs, but also provide useful information for both drugs and diagnostic kits development.

## Graphical abstract

The distributions of mutations and iSNVs in the genomes of SARS‐CoV‐2 VOCs were characterized. The potential correlation between mutations and iSNVs of SARS‐CoV‐2 VOCs was evaluated. Analysis results of both mutations and iSNVs provided some helpful implications to SARS‐CoV‐2 detection and therapeutics.

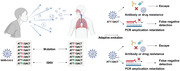

## 1 INTRODUCTION

It has been over 2 years since COVID‐19 became the pandemic. As severe acute respiratory syndrome coronavirus 2 (SARS‐CoV‐2) rapidly and continuously evolves, more than 50,000 lineages had been classified (https://cov‐lineages.org/).^1^ Among these lineages, three variants of concern (VOCs), Alpha (B.1.1.7 and Q.x), Delta (B.1.617.2 and AY.x), and Omicron (B.1.1.529 and BA.x), were dominant in different stages of the pandemic and caused millions of infections. The current global spread VOC (Omicron variant) has a reduced binding capability to many antibodies because of the heavy mutations, such as S375F, N501Y in Spike protein.^2^ With the ongoing evolution of the virus, whether the current PCR detection primers, vaccines, or antiviral agents are effective has become urgent concern. Currently, many studies focus on the mutations landscape to evaluate the impact of mutations on viral infectivity, antigenicity, genomic diversity, and the effectiveness of antibodies, vaccines, or diagnosis kits against the variants.^3^, ^4^, ^5^, ^6^ However, the landscape of the mutations was constructed based on the previous genome data, which lag behind the constantly evolving virus. It is necessary to understand the adaptively evolutionary features of current virus so that the variable regions in its genome can be determined and the efficacy of vaccines or antiviral agents can be confidently evaluated.

RNA viruses have been evolutionarily sharp tuning the replication fidelity to balance the genetic diversity and stability.^7^, ^8^ The replication of RNA viruses with low fidelity is related to the rapid evolution and the adaptation to constantly changing environmental pressures.^9^ Therefore, RNA viruses often generate a large number of minor mutations within the host. Some of these intra‐host mutations have a positive impact on the virological function.^10^ After a period of time, these positive minor mutations can change into the major mutations by competing with other strains.^11^, ^12^ Thus, the intra‐host diversity of RNA virus is usually looked as a key marker for understanding the adaptive evolution of the viruses within the host,^13^, ^14^ and it is often characterized by intra‐host single‐nucleotide variations (iSNVs) or minor alleles.^15^ However, millions of SARS‐CoV‐2 genome sequences available from the public database disguised the minor mutations spectra within the host, limiting our understanding of the intra‐host evolution of SARS‐CoV‐2. Currently, there are some large‐scale studies on the SARS‐CoV‐2 within‐host genomic diversity.^11^, ^15^ For example, Lythgoe et al. performed large‐scale sequencing and found that most mutations generated from virus in the early stage of the pandemic were lost, and few mutations were fixed.^16^ And Tonkin‐Hill et al. also found that the within‐ and between‐host diversities were under a strong purifying selection in the early variants.^17^ However, as the pandemic continues and the novel variants such as Omicron appear, it is meaningful to compare the distribution of the minor mutations in different SARS‐CoV‐2 variants to provide a new insight to variants’ adaptive evolution. Up until now, there is always a certain SARS‐CoV‐2 VOC that dominates the global transmission in different time periods. Investigating the distribution of the intra‐host variation in the genomes of different VOCs would not only help us better understand the adaptive evolution of the widespread SARS‐CoV‐2 variants but also assist us to evaluate the effectiveness of diagnosis kits, vaccines, and drugs against the variants in the future.

In this study, we performed large‐scale investigations to characterize the distributions of both mutations and iSNVs in the genomes of SARS‐CoV‐2 VOCs. The S and N genes were found most variable in the genomes of Omicron variant, which may accelerate the adaptive evolution of this variant. Therefore, some vaccines and PCR primers targeting these two genes are at a high risk of being invalid or showing reduced efficiency in the future. On the contrary, NSP8 gene might be an ideal therapeutic target because of the lowest frequency of mutations and iSNVs in this gene. Our findings not only provide useful information to understand the adaptive evolution of SARS‐CoV‐2 VOCs, but also find some conserved genes, which might be better targets for novel vaccines or drugs development.

## 2 RESULTS

### 2.1 Summary of the datasets used for analysis

Public high‐throughput sequencing raw data (*n* = 1744) of the samples from COVID‐19 patients were collected and used for iSNVs analysis. These data included eight independent SARS‐CoV‐2 genomics sequencing datasets, two of which belonged to our previous work,^18^, ^19^ and other six datasets were downloaded from the public database NCBI and EBI with accession numbers: PRJNA633948, PRJNA639864, PRJNA784038, PRJNA817870, PRJEB46969, and PRJEB47264. Among the 1744 sequencing raw data, only 258 samples had the best genome‐wide sequencing quality (total sequencing coverage more than 95%, and each sequenced position had depth over 100×), 1465 samples had low sequencing quality in some regions of their genomes (total sequencing coverage less than 95% or the number of positions with sequencing depth below 100× was greater than 1000), and the rest 21 samples had low coverage and failed to get the whole genomes (total sequencing coverage smaller than 10%). The detailed data are available in the Supporting Information (Table S1), and the summary of the 258 well‐sequenced samples was listed in Table 1. The average sequencing error rate 0.001 was used to determine iSNVs.

**TABLE 1:** Summary of the well‐sequenced SARS‐CoV‐2 samples for iSNV analysis

**Variant**	**Number of samples**	**Source**	**Time**
B.1/A	76	China, Australia	2020.01–2020.03
Alpha	89	Lebanon	2021.02–2021.04
Beta	20	Pakistan, Australia	2021.06–2021.07
Delta	25	Pakistan, Australia	2021.07–2021.11
Omicron	48	South Africa, France	2021.11–2022.02

Besides, another 3,668,205 high‐quality genomes (genome length >29 kbp, number of unknown bases <30, and number of degenerate bases <70) of SARS‐CoV‐2 from GISAID were used to analyze the distribution of mutations and unique mutations specific to each SARS‐CoV‐2 VOC (Table S2).

### 2.2 Characterization of iSNVs and common mutations in SARS‐CoV‐2 VOCs

The well‐sequenced 258 NGS raw data were selected for iSNVs screening. In summary, we calculated and sorted the frequencies of four bases at each base site of the genome. The base with the second highest frequency is seen as the potential iSNV (minor allele) at the site. Only the qualified minor alleles with the minor allele frequency (MAF) value larger than 0.02, which is about 20 times larger than the sequencing error rate, was considered as the iSNVs. The detailed identification of iSNVs screening can be seen in the method selection.

**FIGURE 1:**
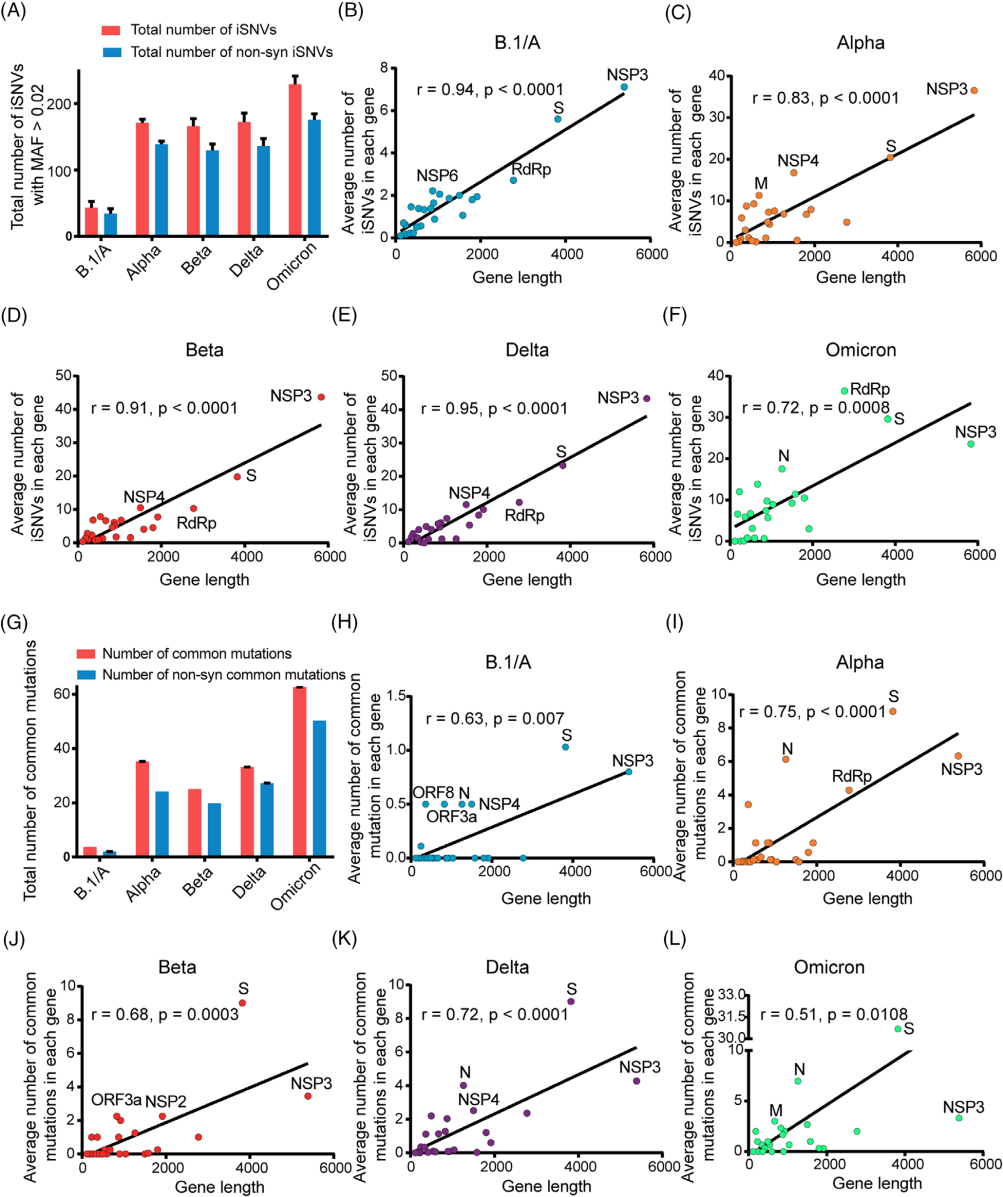
Distribution of iSNVs and mutations in SARS‐CoV‐2 variants. (A) Total number of iSNVs and non‐synonymous (non‐syn) mutated iSNVs with minor allele frequency (MAF) larger than 0.02 in each variant. (B–F) Correlation analysis between iSNV number and gene length in different variants of concern (VOCs) (B: B.1/A, C: Alpha, D: Beta, E: Delta, F: Omicron), the top four iSNV‐rich genes were named in the figures. (G) Total number of common mutations and non‐syn common mutations in each variant. (H–L) Correlation analysis between mutation number and gene length in different VOCs (H: B.1/A, I: Alpha, J: Beta, K: Delta, L: Omicron), the top four mutated genes were marked. In details, the average number of iSNVs in each gene of SARS‐CoV‐2 VOCs was calculated as the (total number of iSNVs in a certain gene)/(total number of the SARS‐CoV‐2 VOC genomes). And the average number of mutations in each gene of SARS‐CoV‐2 VOCs was calculated as the (total number of mutations in a certain gene)/(total number of the SARS‐CoV‐2 VOC genomes).

As shown in Figure 1A and Table S3, the ancestral lineage (B.1/A) of SARS‐CoV‐2 has the lowest number of iSNVs with an average of 41, while the Omicron variant has the maximum number of iSNVs with the average number of 223. Although most of the iSNVs belong to non‐synonymous (non‐syn) mutations, the results of *K*_a_/*K*_s_ analysis revealed that most iSNVs in the coding regions of SARS‐CoV‐2 variants were under the purifying selection (*K*_a_/*K*_s_ values <1, Table S3 sheet B). Besides, we found that the NSP3 gene had the maximum number of iSNVs in the ancestral lineage (Figure 1B), as it is the longest gene in the SARS‐CoV‐2 genome. Interestingly, the number of iSNVs in individual genes of Alpha, Beta, and Delta variants also correlate to the gene lengths, but all are higher than those of the ancestral lineage B.1/A (Figure 1B–E). However, this correlation was decreased in the Omicron variant because some genes such as S, RdRp, and N have significantly higher number of iSNVs (Figure 1F) than those of the previous VOCs. Generally, among the genes, S and NSP3 genes were the top two iSNVs‐rich genes, while NSP8 and ORF10 genes have the least iSNVs (Table S3 sheet B). More detailed information, such as iSNV number, iSNV density, and *K*_a_/*K*_s_ values are available in Table S3 sheet B.

Furthermore, the common mutations (mutations present in over 50% isolates of a certain SARS‐CoV‐2 lineage) of each SARS‐CoV‐2 variant were identified based on the large‐scale genomes data. As shown in Figure 1 and Table S3, similar to iSNVs, the Omicron variant contains more mutations than other VOCs. Analyzing results of the mutations in different genes showed that S, N, and NSP3 genes were the three most highly variable genes in different VOCs. Further analysis indicated that the mutations in the S gene of SARS‐CoV‐2 VOCs were under the positive selection (*K*_a_/*K*_s_ values >1, Table S3 sheet B). The other genes, such as NSP8 and ORF10 genes, are the most conserved (Table S3 sheet B). RdRp gene has fewer mutations than iSNVs in different VOCs (Table S3 sheet B). More detailed information, such as mutation number, mutation density, and *K*_a_/*K*_s_ values, is available in Table S3 sheet B.

### 2.3 Evaluation of relationship between iSNVs and mutations

**FIGURE 2:**
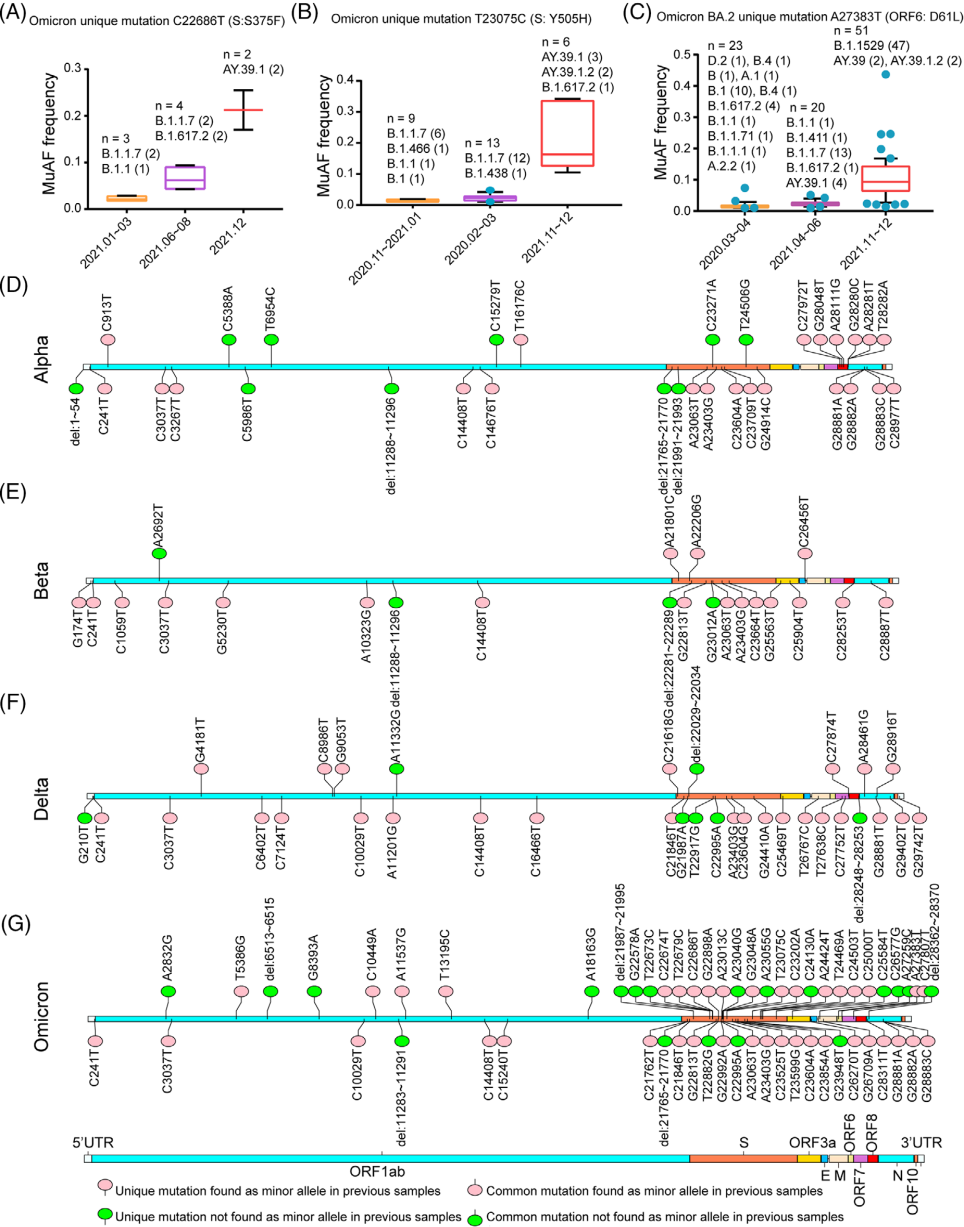
The relationship between iSNVs and mutations. (A–C) The common mutations could be found as minor allele in the previous samples and the relationship between their minor allele frequency and time. The boxplots were plotted with mean value of mutated minor allele frequency (MuAF) and standard deviation. The outliers are shown as scatter points in the boxplots. (D–G) Genome‐wide profiles of whether unique or common mutations were present as minor alleles in previous samples among the SARS‐CoV‐2 variants of concern (VOCs) (Alpha: D, Beta: E, Delta: F, Omicron: G). The unique mutations specific to a certain SARS‐CoV‐2 VOC were marked at the top and the common mutations were marked at the bottom. If a mutation could be found as a minor allele in previous samples, this mutation was marked with pink. Else, it was marked with green.

During analysis of the distributions of iSNVs and common mutations in different VOCs, we found that many common mutations of the SARS‐CoV‐2 variants were present as iSNVs (minor allele) in previous sequencing datasets. For example, two unique mutations C22686T (S375F) and T23075C (Y505H) were in the receptor binding domain (RBD) of Omicron variant and reported to be associated with decreased protein stability and increased risks of infectivity.^20^ As shown in Figure 2A and Table S4, the mutation S375F presented as the iSNV in some Alpha isolates emerged in early 2021, and the mutated minor allele frequency (MuAF) of this iSNV increased over time in some later Alpha and Delta strains. Similarly, the unique mutation Y505H identified as a minor allele in some isolates emerged at the end of 2020. Later, the MuAF of this allele significantly increased in the Delta strains emerged in November 2021 (Figure 2B). Similarly, another unique mutation A27383T (D61L) specific for Omicron sublineage BA.2 was found as a minor allele in the samples isolated from early stage in 2020, and MuAF of this allele finally increased in the Omicron variant B.1.1.529 (Figure 2C). Collectively, many mutations of SARS‐CoV‐2 VOCs, especially 41 mutations of Omicron variant, can be found as minor alleles in previous variant samples such as Alpha and Delta in different countries (Figure 2D–G and Table S4).

### 2.4 Potential contribution of mutations to high intra‐host diversities in SARS‐CoV‐2 VOCs

**FIGURE 3:**
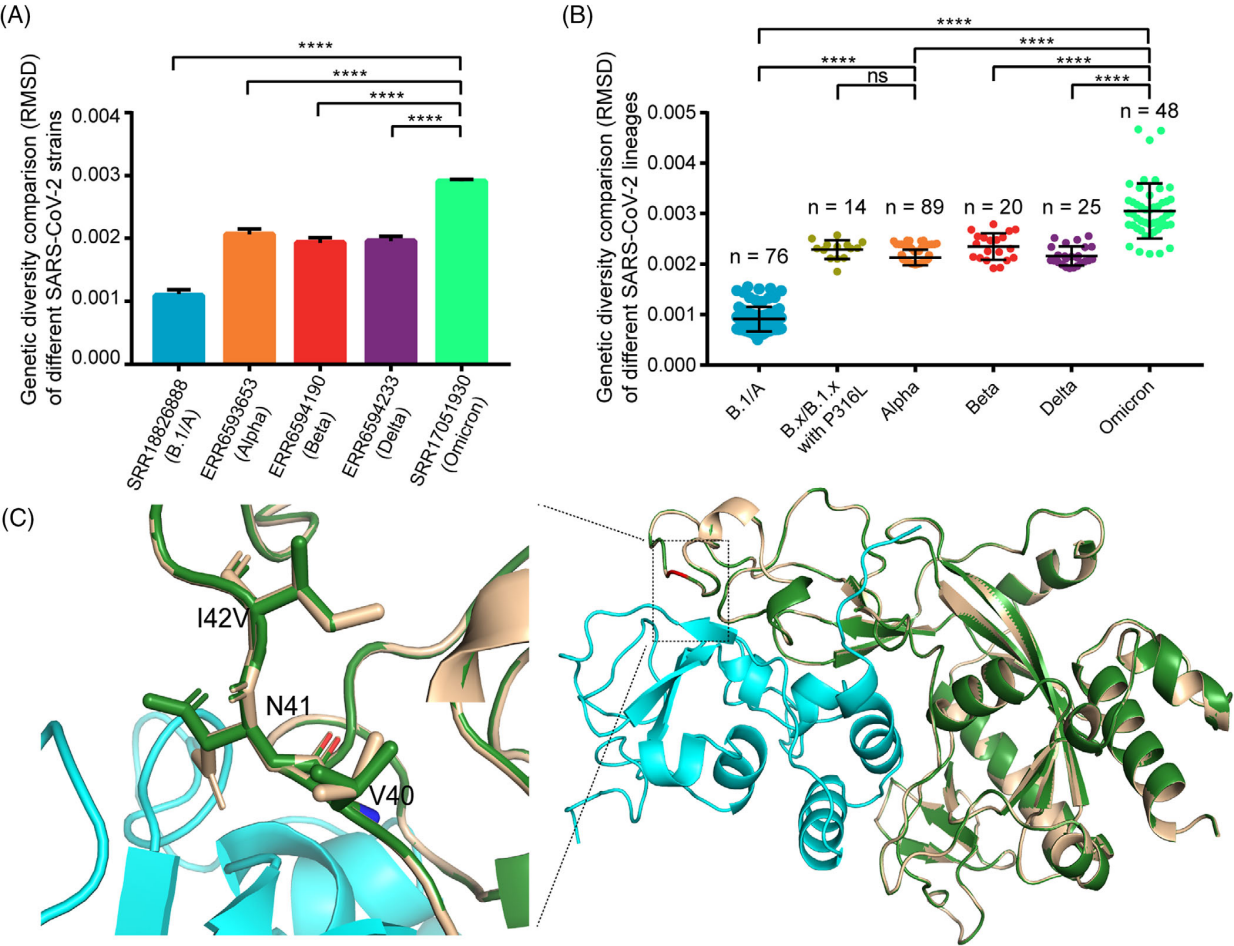
Intra‐host genetic diversity of each SARS‐CoV‐2 variant. (A) Intra‐host genetic diversity at representative SARS‐CoV‐2 strain level. The root‐mean‐square‐deviation (RMSD) is shown with mean values ± SEM. (B) Intra‐host genetic diversity at lineage level. Each scatter represents the average RMSD value of each isolate in that lineage. The number of isolates in each lineage is marked. The significant analysis is calculated by the Kruskal–Wallis test. (C) Molecular dynamics simulations of I42V mutation in NSP14 (PDB accession number: 7DIY). The reference structure of NSP14 (I42) is highlighted with white. The mutated structure of NSP14 (V42) is shown with green. The structure of NSP10 is highlighted with cyan. Amino acid residue conformations affected by the mutation are shown.

We evaluated the intra‐host diversities of the SARS‐CoV‐2 strains by calculating the root‐mean‐square‐deviation (RMSD) value at each site across the genome. Each column in Figure 3A shows the RMSD data at each position across the SARS‐CoV‐2 genome of one representative SARS‐CoV‐2 strain. Figure 3B shows the average RMSD data of all well‐sequenced SARS‐CoV‐2 strains in different lineages (one dot represents one strain). Figure 3A,B indicate that the intra‐host diversities of all SARS‐CoV‐2 VOCs are significantly higher than that of the ancestral strain or lineage (Kruskal–Wallis test, *p*‐values <0.0001). Omicron variants have the highest diversity among the analyzed VOCs, irrespective of the individual strain level or lineage level.

Previous studies reported that RNA‐dependent RNA polymerase (RdRp) and NSP14 are two major contributors to the intra‐host diversities of coronaviruses,^9^, ^11^ because the product of NSP14 gene has 3′ to 5′ exonuclease activity that can proofread the mismatched nucleotides replicated from RdRp. Therefore, we further analyzed whether any mutations in RdRp and NSP14 genes could contribute to the increased diversities of SARS‐CoV‐2 VOCs. According to the large‐scale genomes analysis, Omicron variant has two non‐synonymous mutations, C14408T (P316L) in RdRp and A18163G (I42V) in NSP14, but the other three VOCs only have one non‐synonymous mutation C14408T (P316L) in RdRp (Figure 2 and Table S2). C14408T (P316L) is the second hotspot mutation after the mutation A23403G (D614G). To investigate whether the mutation P316L in RdRp could affect the genetic diversity of SARS‐CoV‐2 VOCs, other 14 deep‐sequenced non‐VOC samples in the early stage of the pandemic that belonged to B.1.*x* or B.*x*, but with P316L mutation in RdRp and a few mutations in other regions, were included to calculate intra‐host diversities (Table S5). As shown in Figure 3B, the RMSD values showed that the intra‐host diversities of these 14 samples were also significantly higher than that of the ancestral lineage but close to those of Alpha variants (Kruskal–Wallis test, *p*‐value <0.0001). One thing to be noticed is that B.1.*x* or B.*x* lineages with P316L mutation only contain a few mutations (Table S5) compared to the reference genome (MN908947.3). Besides the hotspot mutation P316L, Omicron variants have the specific mutation A18163G (I42V) in NSP14 gene (Figure 2G and Table S2). Owing to that the proofreading activity of NSP14 depends on the binding capability of NSP10,^21^ and I42V is close to the key sites that directly interact with NSP10,^22^ molecular dynamics simulation was performed to compare the changes of the binding free energy before and after the mutation. Using the CryoEM structure (PDB accession number: 7DIY), we found the binding free energy is −36.62 kcal/mol before mutation, but it changed to −31.53 kcal/mol after the mutation, which means that the binding between NSP14 and NSP10 may become weaker after the mutation, leading to weaker proofreading activity (Figure 3C).

### 2.5 Impact of mutations and iSNVs on RT‐PCR detection

**FIGURE 4:**
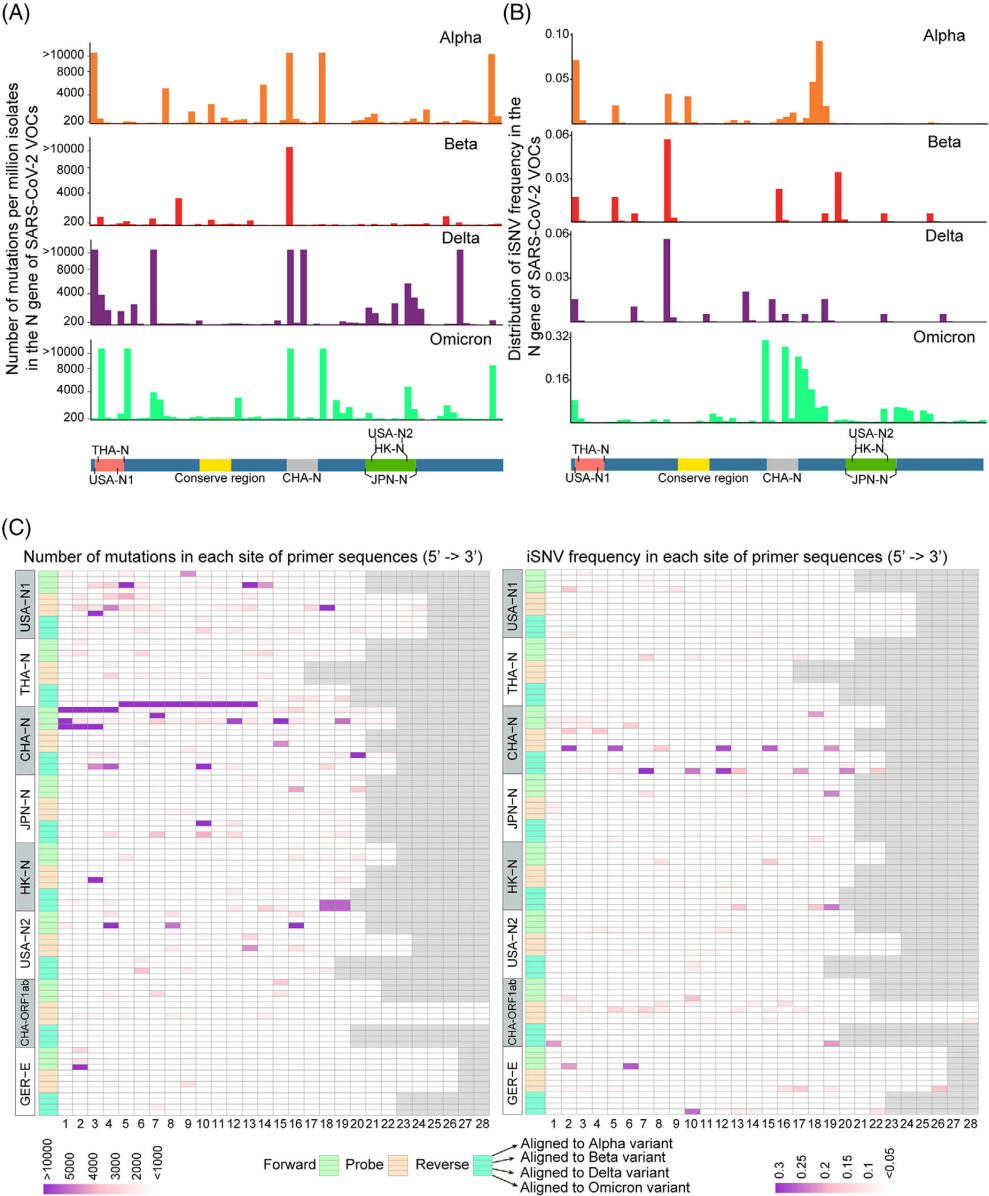
Distribution of mutations and iSNV frequency in the N gene of SARS‐CoV‐2 variants of concern (VOCs). (A) Distribution of mutations in the N gene of SARS‐CoV‐2 VOCs. (B) Distribution of iSNV frequency in the N gene of SARS‐CoV‐2 VOCs. The most conserved region and the amplification regions of the primer probe sets targeting N gene from the Center for Disease Control and Prevention (CDC) of several areas were also highlighted. (C) Mutations (left) and iSNV frequency (right) in each position of several primer probe sets announced by some CDCs worldwide. All sequences were 5′‐aligned, and the short sequences were filled with the gray squares at their 3′ end to match the length with the longest sequence. The numbers 1–28 represent the sites in primer/probe. Collectively, in order to facilitate visualization, the number of mutations larger than a certain value was visualized by this value. And the iSNV frequency in each VOC was calculated as (total number of iSNVs in the sites)/(total number of the well‐sequenced samples of a certain VOC). The full names of the abbreviations for countries/areas are: Thailand (THA), China (CHA), United States of America (USA), Japan (JPN), Hong Kong Special Administrative Region (HK), and Germany (GER). The CHA‐ORF1ab represents the primer probe of China CDC targeting ORF1ab region and the GER‐E represents the primer probe of Germany CDC targeting E gene.

The current gold‐standard approach for SARS‐CoV‐2 detection is RT‐PCR. One of the obvious factors to keep the effectiveness of these assays is that the oligonucleotide primers and probes can accurately match the target sequence of different variants. Mutations, especially the mutations in the probe or at 3′ end of the primers, may lead to invalid PCR assays. We further analyzed whether mutations and iSNVs in N gene, which was chosen mostly as the target gene for detection because of its high abundance lead by the complex numerous discontinuous transcription events,^23^ might affect the PCR assays widely used. As shown in Figure 4, almost all the primer probe sets matched regions had thousands of mutations, but most of these mutations were far away from the key sites that could affect PCR amplification, except for the primer probe sets from Thailand and Hong Kong CDCs. There is a deletion in the N gene of Omicron variants (Table S2) in the region of the reverse primer from Thailand CDC and thousands of genomes have mutations in the 3′ end of the reverse primer from Hong Kong CDC (Figure 4C). The widely used primer probe set from China CDC was located in the iSNVs‐rich regions of N gene (Figure 4B,C). In contrast, two primer probe sets targeting ORF1ab and E gene from China CDC and Germany CDC, respectively, are found much conserved (Figure 4C). Moreover, there is one most conserved region (from 28595 to 28702 in the genome of MN908947.3) in the N gene, which may be used for designing new primer probe sets for PCR detection of SARS‐CoV‐2.

### 2.6 Implications of mutations and iSNVs on vaccines

The S protein of SARS‐CoV‐2 majorly interacts with the host angiotensin‐converting enzyme 2 (ACE2) receptor before entering a cell to initiate the infection, so it was mainly used as the target for vaccine development. With the circulation of the SARS‐CoV‐2 variants, most monoclonal antibodies approved by FDA such as bamlanivimab, etesevimab, casirivimab, imdevimab, tixagevimab, and cilgavimab were reported to have reduced efficacies against Omicron variants because of the heavy mutations in RBD region.^20^ We further evaluated the potential impact on the vaccine effectiveness based on the mutations and iSNV landscapes in S gene.

**FIGURE 5:**
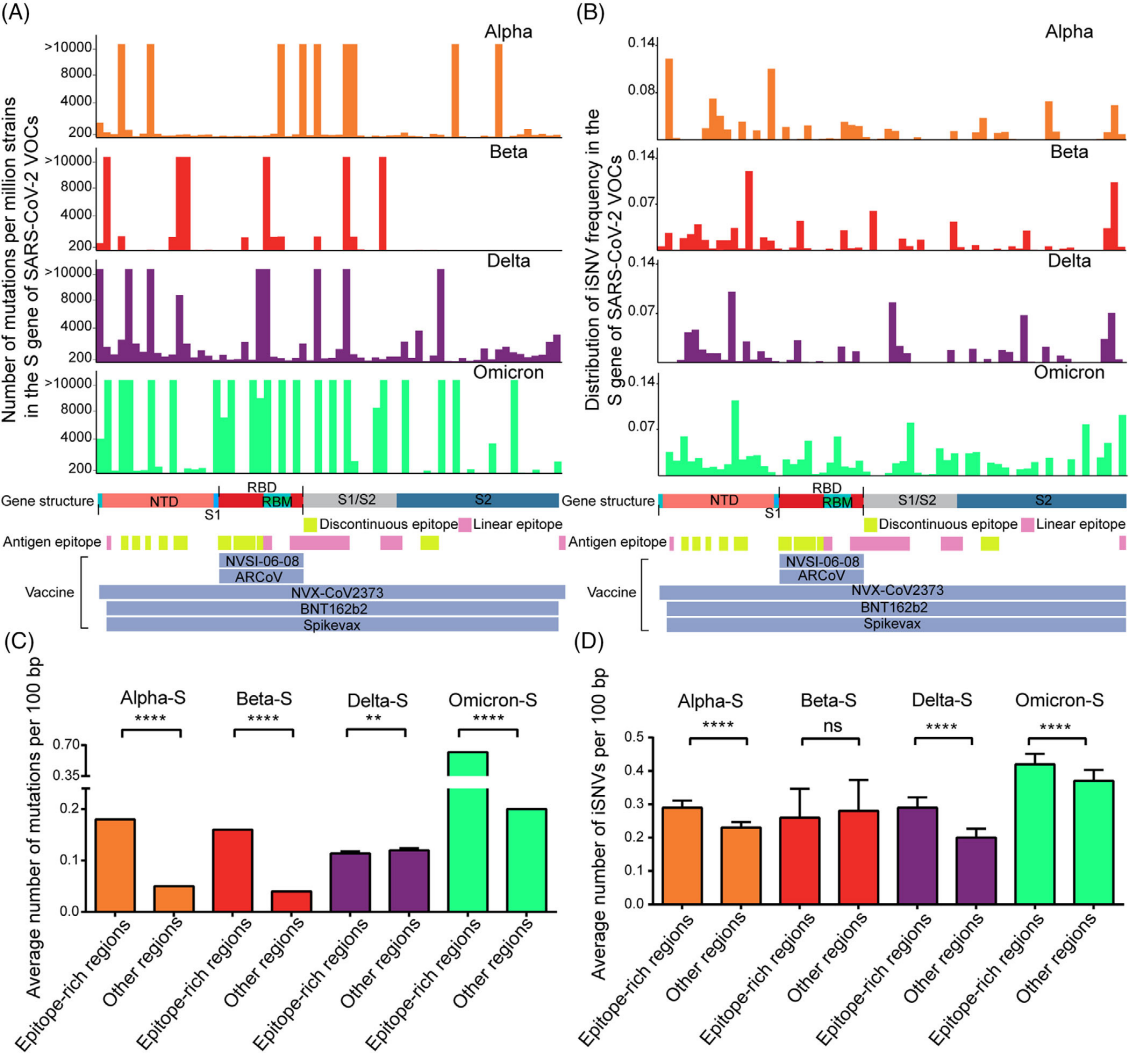
Distribution of mutations and iSNV frequency in the S gene of SARS‐CoV‐2 variants of concern (VOCs). (A) Distribution of the mutations in the S gene of SARS‐CoV‐2 VOCs. (B) Distribution of the iSNV frequency in the S gene of SARS‐CoV‐2 VOCs. (C and D) Comparison of iSNVs and mutations distribution in the epitope‐rich regions and other regions in the S gene of each VOC. The significant difference was calculated by the Kruskal–Wallis test. In details, the iSNV frequency in each VOC was calculated as (total number of iSNVs in the site)/(total number of the well‐sequenced samples of a certain VOC), and the antigen epitopes including discontinuous epitopes and linear epitopes were referred to the results from http://tools.iedb.org/ and Li et al.^56^ The vaccine sequences were aligned to the reference S gene and highlighted at the bottom of the figure.

As shown in Figure 5, the N‐terminal domain (NTD) and RBD regions contained many variable sites, and Omicron variant has much higher densities of both mutations and iSNVs. Even worse, most variants, especially Omicron, have more mutations and iSNVs in the epitope‐rich regions than that of other regions (Figure 5C,D, most *p*‐values <0.0001 by Kruskal–Wallis test). Therefore, it is highly possible that efficacy of vaccines targeting S gene is variable to different VOCs. For example, two mRNA vaccines named BNT162b2 and Spikevax approved by FDA, another mRNA vaccine named ARCoV under phase III clinical trials, and two other subunit vaccines, NVSI‐06‐08 in clinical trials and NVX‐COV2373 for Emergency Use Authorization (EUA) by FDA (https://www.fda.gov/emergency‐preparedness‐and‐response/coronavirus‐disease‐2019‐covid‐19/covid‐19‐vaccines),^24^, ^25^ which covered the whole RBD region or the full length of S gene (Figure 5), were designed based on sequences of the ancestral strains or Beta and Kappa variants, other than current Omicron variants.

### 2.7 Implications of mutations and iSNVs on antiviral drugs

**FIGURE 6:**
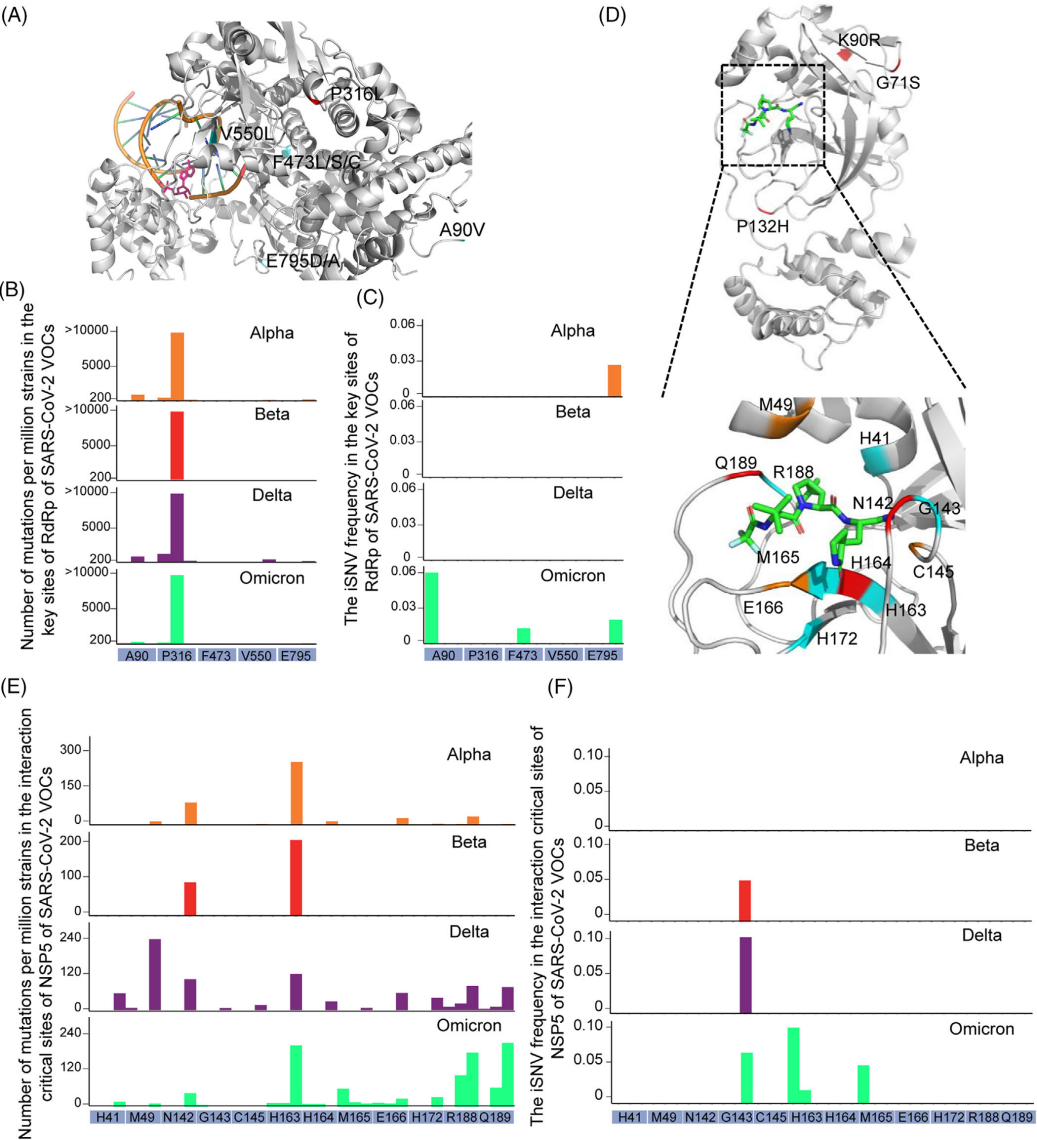
Distribution of mutations and iSNV frequency in the RdRp and NSP5 genes. (A) Structure of RNA‐dependent RNA polymerase (gray) interaction with the antiviral drug remdesivir (pink). The reported five mutations that can affect the remdesivir resistance of RdRp are highlighted in the structure (A), and the number of mutations and iSNV frequency of these sites are shown in (B) and (C). (D) Structure of the main protease (NSP5, gray) interacting with the antiviral drug paxlovid (green). The interaction‐critical sites with paxlovid of NSP5 are highlighted in the structure (D). The number of mutations and iSNV frequency of these sites are shown in (E) and (F). The number of mutations larger than a certain value was visualized by this value. The iSNV frequency in each variants of concern (VOC) was calculated as (total number of iSNVs in the sites)/(total number of well‐sequenced samples of a certain VOC).

Most of the antiviral drugs target the products of ORF1ab region, where few mutations in SARS‐CoV‐2 VOCs are included (Figure 1G and Table S3 sheet B). Currently, remdesivir is the only drug approved by FDA, and Chinese scientists have developed an oral remdesivir derivative VV116.^26^, ^27^ Another drug was approved for EUA by FDA named paxlovid targeting the main protease (NSP5).^28^ For the antiviral drug remdesivir, there are five reported mutations (A90V, P316L, F473L/S/C, V550L, and E795D/A) that could affect the affinity of remdesivir and RdRp.^29^ Among them, the mutation C14408T (P316L) was found capable of increasing the affinity of remdesivir and RdRp.^29^ The mutations and iSNVs profiles of RdRp revealed that except the common mutation P316L shared by SARS‐CoV‐2 VOCs, the key sites of RdRp to remdesivir were extremely conserved (Figure 6B,C). For another drug paxlovid, the common mutations G71S (Alpha), K90R (Beta), and P132H (Omicron) were far away from the interaction‐critical sites of the drug (Figure 6D) with NSP5. And there were few isolates with mutations and iSNVs in the interaction‐critical sites (Figure 6E,F).^28^ Compared with the variations in S and N genes, genes targeted by the antiviral drugs have much less mutations and iSNVs.

## 3 DISCUSSION

Previous studies on other RNA viruses, such as yellow fever virus and influenza virus, revealed that the intra‐host diversity could provide useful information on virus adaptive evolution and host‐to‐host transmission during the epidemic.^13^, ^14^ In our study, we compared iSNVs and mutation features of SARS‐CoV‐2 VOCs during different stages of COIVD‐19 pandemic. Compared with the previous studies,^11^, ^16^, ^17^, ^30^ some interesting findings were obtained, which may deepen our understanding of the adaptive evolution of SARS‐CoV‐2 VOCs, especially Omicron variant. The distribution of iSNVs (Figure 1A–F) showed that VOCs have more iSNVs than the ancestral lineage. The selection pressure analysis showed most genes in the genomes of SARS‐CoV‐2 VOCs were under the purifying selection, which is consistent with the findings of Lythgoe et al.^16^ But we found that Omicron variant is quite special with much higher iSNVs. Especially the S gene of Omicron variant was under positive selection, which indicated that S gene may be in the process of fast evolution. We further found that the mutation C14408T in RdRp gene may contribute to the increased intra‐host diversities of all the SARS‐CoV‐2 VOCs, which matched the studies of Pathak et al. and Pachetti et al.,^11^, ^31^ and the mutation A18163G in NSP14 gene might contribute further to the even higher diversities of Omicron variants, as A18163G in NSP14 is the unique mutation specific for Omicron variant (Table S2) and did not present in previous variants. It is possible that this mutation might affect the binding capability between NSP14 and NSP10, leading to a weaker proofreading activity (Figure 3C), because NSP14 can proofread the mismatch produced by RdRp after binding to NSP10.^21^, ^22^ However, further experimental confirmation is needed.

Overall, the iSNV results indicated that the VOCs have adapted to humans easier than the ancestral strain during spread and the higher iSNVs of Omicron variants may contribute to their much higher transmission rates. We also compared our results to the deep‐sequencing NGS data of the B.1/A lineage submitted by UK scientists in the early stage of the pandemic.^17^ The RMSD results revealed that the intra‐host genetic diversities of the B.1/A variants in the early stage of the pandemic in the United Kingdom were low, which is consistent with our conclusions (Figure S1). At the same time, VOCs have obtained more and more mutations during spread (Figure 1G–L), which led to concerns about the effectiveness of current vaccines and detection using current PCR primers. For example, the N gene was often seen as the PCR target, but it was mutable because of the accumulation of C‐containing codons in its tether region.^32^, ^33^ In this study, we found that some mutations in the key sites targeted by the CDC PCR assays may lead to invalid detection. And other mutations, especially the mutations in S gene, may lead to immune evasion of the virus to vaccines and antibodies generated in previous infections. As the current pandemic is still ongoing, it highlights the importance of developing effective detection primers, vaccines and drugs to combat the widespread virus. As we identified that the distributions between mutations and iSNVs are similar and some mutations could be traced to iSNVs in previous variants, it may be useful to track the distributions of iSNVs in current VOCs to avoid the variable regions in the genome during designing primer probes and vaccines. Unlike S gene, the key sites of the RdRp and NSP5 genes targeted by current antiviral drugs are quite conserved for all the VOCs, which may mean that the current antiviral drugs are still effective for the time being. Our results also revealed that NSP8 gene, which works as a key and essential cofactor for RNA replication and synthesis,^34^ was one of the extremely conserved genes and could be considered as a therapeutic and detection target also.

Although the overall distributions of iSNVs and mutations are similar, there are still some iSNVs‐rich genes with low mutation frequency such as RdRp gene (Figure 1). The fitness cost of mutation is one of the important factors that determines whether a minor mutation can be retained. The RNA‐dependent RNA polymerase is an essential enzyme that helps RNA synthesis and replication of SARS‐CoV‐2 genome. Therefore, proper and efficient synthesis of viral RNA is important for virus survival.^35^ However, the fitness costs of mutations in RdRp are very high. For example, Agostini et al. found that the mutation V533L or F476L in RdRp could reduce the susceptibility of coronavirus mouse hepatitis virus (MHV) to the antiviral drug remdesivir, while the replication ability of the mutated virus was significantly decreased and failed to compete with wildtype.^36^ These two mutations corresponded to V550L and F473L in RdRp of SARS‐CoV‐2, and the corresponding mutation rate was also extremely low (Figure 6A–C). Thus, accumulation of mutations in RdRp might probably damage the virus and limit the emergence of mutations. And the *K*_a_/*K*_s_ values of iSNVs and mutations in RdRp were smaller than 1, which means the deleterious mutations in this gene might be eliminated (Table S3 sheet B). On the contrary, the Spike protein is located on the surface of SARS‐CoV‐2 and it can mediate viral entry by binding with the host cell surface protein ACE2. Thus, the Spike protein becomes the target of most neutralizing antibodies and vaccines. The mutations in Spike protein, especially in the antigen epitope regions, are favorable for the virus to escape, neutralizing antibodies or enhancing the receptor binding capability.^37^ Hence, in the scenario of mass vaccination of different vaccines, the iSNVs in S gene can favor the virus survival and have large probability to be accumulated, and the *K*_a_/*K*_s_ value of this gene larger than 1 also supports this hypothesis (Table S3 sheet B). It might be the reason why the Omicron variant has the cluster mutations and iSNVs in its S gene (Figure 5).^38^

Our results also indicated that many mutations of SARS‐CoV‐2 VOCs that may be traced as minor alleles in previous VOCs emerged in different countries and the early iSNVs provide a mutational pool shaping the global evolution of variants, which are consistent to the previous studies by Pathak et al.^11^ and Li et al.^30^ But we also found there were some mutations that cannot be traced to previous iSNVs, which might be partially due to the limitations of the collected datasets. However, as the variants widely spread, they probably underwent multiple ways to generate the mutations, such as different host selection, environmental mutagenesis, or recombination.^39^, ^40^ For example, Bate et al. found that the unique mutation A23055G (Q498R) of Omicron variant combined with A23063T (N501Y) allowed the virus to bind tightly to rat ACE2,^41^ and the mutation Q498R was not found as minor allele in the previous isolates (Figure 2G). It was suggested that the evolution of SARS‐CoV‐2 in the past might have gone through a highly complex process, such as zoonotic transfer from animal to human, and human‐to‐animal‐to‐human.^42^

In summary, we characterized the mutation and intra‐host variation landscapes of typical SARS‐CoV‐2 VOCs. Our findings not only suggested that Omicron variant is still under a high degree of adaptive evolution, but also identified two mutations, which might be associated with the increased intra‐host diversity. Further analyzing mutations and iSNVs in important genes revealed that some PCR primer probe sets need to be re‐designed to avoid invalid detection for certain variants, and vaccines targeted to S gene need to be updated so that they are efficient against new variants. However, the genes targeted by current antiviral drugs are quite conserved, which might mean that those drugs are active to current VOCs and future variants for only short time.

## 4 MATERIALS AND METHODS

### 4.1 Next‐generation deep sequencing data collection and processing

From our previous study,^18^ the third‐generation sequencing platform still has false‐positive minor mutations caused by the technical errors during sequencing. Therefore, only next‐generation pair‐end deep sequencing data meet the requirements in intra‐host variation calling. Besides, each independent NGS dataset should contain at least 100 COVID‐19 patient samples. And we also ensured that different sequencing datasets came from the patients in different countries at different times. Finally, a total of eight independent NGS datasets were selected in this study. Among them, two datasets belonged to our previous work,^18^, ^19^ and the other six datasets were collected from the public databases NCBI and EBI. The accession numbers of the six studies are PRJNA633948, PRJNA639864, PRJNA784038, PRJNA817870, PRJEB46969, and PRJEB47264. Total 1744 sequencing data from 1744 SARS‐CoV‐2‐positive nasopharyngeal swab samples, covering different areas worldwide from 2020 to 2022, as listed in Table S1, were used to intra‐host genetic diversity analysis.

The raw data in FASTQ format were downloaded. The low‐quality reads were trimmed by the Trimmomatic software (version 0.36).^43^ Clean reads were then aligned to the SARS‐CoV‐2 reference genome (accession number: MN908947.3) via BWA software (version 0.7.17) with the default options.^44^ The coverage and sequencing depth were calculated by the samtools software.^45^ Only samples with high sequencing coverage (>95%) and enough depth (>100) were used for downstream analysis.

### 4.2 Lineage typing of SARS‐CoV‐2 isolates

After mapping the clean reads to the SARS‐CoV‐2 reference genome, the viral reads were extracted by the bam2fastq software (version 1.1.0, https://genome.sph.umich.edu/wiki/BamUtil:_bam2FastQ). The de novo assembly was performed by the megahit software (version 1.2.9) based on the viral reads.^46^ Meanwhile, the point mutations, deletions, and insertions of each sequence were identified by the SnpEff software (version 5.1).^47^ The completed or nearly full length of SARS‐CoV‐2 genomes was uploaded to the online sever https://pangolin.cog‐uk.io/ to calculate the lineages. Based on the lineage information, the taxonomy of SARS‐CoV‐2 variant could be accurately determined.

### 4.3 Calling of intra‐host single‐nucleotide variations

The iSNVs are defined as the allele with minor frequency co‐existing with the reference allele (major frequency) at the same position within the same sample. The identification of iSNVs was basically executed by our established pipelines and referred to Nicole's work.^9^, ^48^ In details, the jellyfish software (version 2.3.0) was utilized to evaluate the sequencing errors of each NGS data.^49^ In order to determine the iSNVs more strictly, three requirements should be met: (a) sequencing depth greater than 100; (b) MAF should be larger than 0.02, which is significantly greater than the average sequencing errors rate (0.001); (c) the position of each iSNV was supported by the inner part of read (excluding 10 bp on each end).

### 4.4 Genetic diversity analysis

As mentioned in Rozen‐Gagnon's publication, the root mean square deviation (RMSD) values of the variance at each nucleotide position were used to estimate the viral genetic diversity within a deep sequencing sample.^50^ Each position was considered by the RMSD value of genetic diversity across the genome.^51^ Therefore, in this study, we used the RMSD value to evaluate the intra‐host genetic polymorphisms of differential SARS‐CoV‐2 variants based on the genome‐wide minor allele profile generated from the above description.

### 4.5 Large‐scale genomics data analysis for the mutations

A total of 3,668,205 SARS‐CoV‐2 completed high‐quality genomes were downloaded from the GISAID database (https://www.gisaid.org/) covering all isolates that emerged from 2019 to April 2022. These genomes were selected based on the criteria: (a) genome length is longer than 29 kbp, (b) the unknown base(s) are less than 30, and (c) the degenerate base(s) are less than 70. These large‐scale genomics data were used to construct the mutations landscapes and identify unique mutations specific to SARS‐CoV‐2 VOCs according to our established pipelines.^52^ In particular, our established pipelines performed pair‐wise alignment between each SARS‐CoV‐2 genome and the reference genome (MN908947.3). After the alignment, our pipeline can identify the mutations of each isolate against the reference genome. Combined with the lineage information, the mutation coverage of a certain SARS‐CoV‐2 lineage can be calculated by (total number of genomes identified with this mutation from this lineage)/(total number of genomes from this lineage). If a mutation presented in over 50% of the corresponding lineage, it is considered as the common mutation of this lineage. If a common mutation only occurred in a certain SARS‐CoV‐2 lineage, this mutation can be considered as the unique mutation specific to this lineage.

### 4.6 Molecular dynamics simulations

The molecular dynamics simulations were used to evaluate whether a single amino acid substitution on NSP14 would affect the binding capability between the two nonstructural proteins NSP10 and NSP14. The high‐resolution CryoEM result of NSP14–NSP10 complex (7DIY) from Protein Data Bank (PDB) database was used for amino acid substitution prediction. The Chimera software^53^ was executed to minimize the energy of the mutated protein structure to adjust the changes of the amino acid and its surrounding amino acid structure after mutation. The binding free energies for NSP14 to NSP10 were calculated by the HawkDock sever based on the Molecular Mechanics/Generalized Born Surface Area (MM/GBSA) model.^54^

### 4.7 Selection pressure calculation

The selection pressures of iSNVs and mutations were calculated by the software KaKs_calculator (version: 3.0).^55^

### 4.8 Statistical analysis

All the statistical analyses were performed in the GraphPad Prism 7 software (GraphPad Inc., San Diego, CA, USA) or the R program language (version: 3.6.3, https://cran.r‐project.org/bin/windows/base/old/3.6.3/). Before statistical analysis, the distribution feature of each data was judged. Collectively, in this study, the independent groups were analyzed by Kruskal–Wallis test and the correlation coefficient was calculated by Pearson correlation analysis. Data were showed as mean ± SEM. Statistical differences were considered significant at *p < 0.05, **p < 0.01, ***p < 0.001, and ****p < 0.0001.

## AUTHOR CONTRIBUTIONS

Dongyan Xiong designed experiments, performed all bioinformatics analysis, analyzed the experiment results, and drafted the manuscript. Xiaoxu Zhang attended the results discussion. Junping Yu designed experiments, attended the results discussion, and revised the manuscript. Hongping Wei generated the idea, designed experiments, finalized the manuscript, and provided funding. All authors have read and approved the final manuscript.

## CONFLICT OF INTEREST

The authors declare that they have no conflicts of interest.

## ETHICS STATEMENT

No research activity for this manuscript requires an ethics approval.

## Supporting information

Supporting Information

Supporting Information

Supporting Information

Supporting Information

Supporting Information

Supporting Information

## Data Availability

The commands used in pipeline are available at the following GitHub repository: https://github.com/MisgaXiong/Sripts_for_Paper/tree/master/SARS‐CoV‐2‐mutation. The data that support the findings of this study are available in the Supporting Information of this article.
