# Supplementary material for: Distribution of intra‐host variations and mutations in the genomes of SARS‐CoV‐2 and their implications on detection and therapeutics
Source: MedComm (2020). 2022 Dec 2;3(4):e186. doi: 10.1002/mco2.186 (PMC9717708; doi:10.1002/mco2.186)
Supplement: Supplementary file 1 — Supporting Information [file MCO2-3-0-s003.docx]

**Supplementary materials**

**Distribution of intra-host variations and mutations in the genomes of SARS-CoV-2 and their implications to detection and therapeutics**

Dongyan Xiong^1,2^, Xiaoxu Zhang^1^, Junping Yu^1,2*^, Hongping Wei^1,2*^

*1. CAS Key Laboratory of Special Pathogens and Biosafety, Center for Biosafety Mega-Science, Wuhan Institute of Virology, Chinese Academy of Sciences, #44 Xiaohongshan, Wuchang, Wuhan, 430071, China;*

*2.* *University of Chinese Academy of Sciences, Beijing, 100049, China;*

For correspondence:

All correspondence should be sent to Junping Yu [yujp@wh.iov.cn](mailto:yujp@wh.iov.cn), Tel: 86-27-87998263, or Hongping Wei [hpwei@wh.iov.cn](mailto:hpwei@wh.iov.cn), Tel: 86-27-87998873


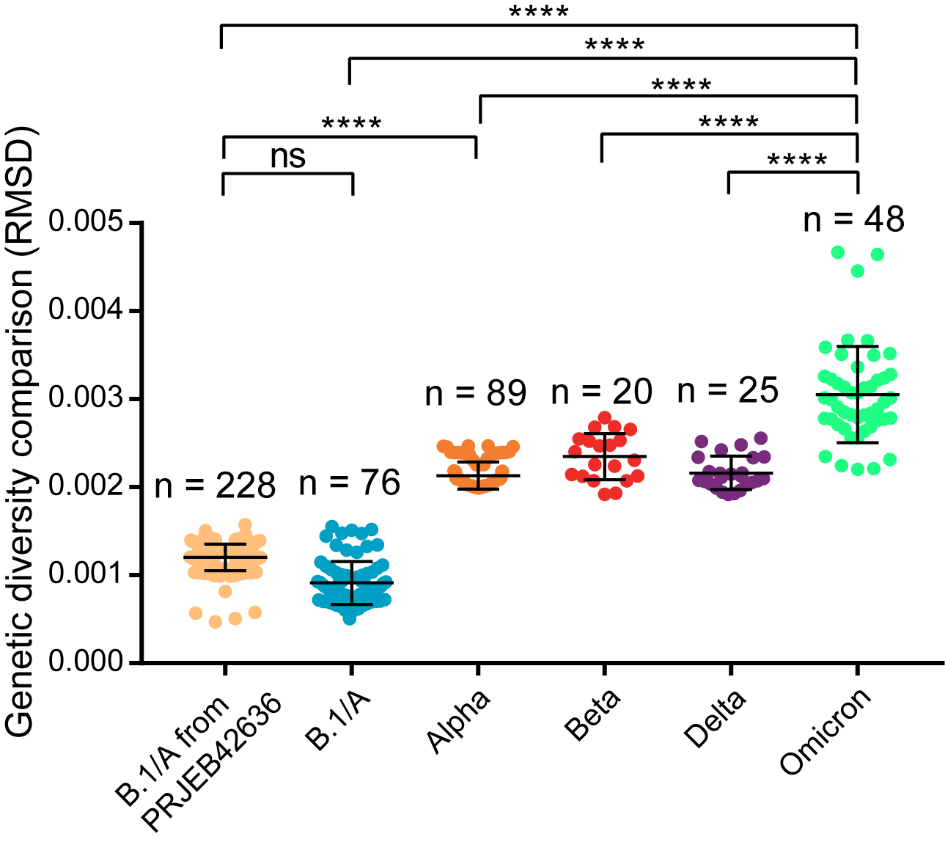


**Figure S1**: Genetic diversity comparison of SARS-CoV-2 lineages including public data of EBI accession: PRJEB42623 from the study of Tonkin-Hill, G. et al^1^. There were 228 sequencing data among 1,181 SARS-CoV-2 strains emerging in United Kingdom in early stage of the pandemic satisfied our standards (total sequencing coverage lager than 95% and each sequenced position had depth over 100×). All of these 228 isolates belong to B.1/A lineage. Each scatter represents the average RMSD value of each isolate. The significant analysis was calculated by the Kruskal-Wallis test. The results of the 228 isolates were consistent with our results showed in Figure 3B.

**Reference**

1. Tonkin-Hill G, Martincorena I, Amato R, et al. Patterns of within-host genetic diversity in SARS-CoV-2. *eLife*. 2021;10
